# Genome-Wide Identification and Expression Analysis of the Ascorbate Oxidase Gene Family in *Gossypium hirsutum* Reveals the Critical Role of *GhAO1A* in Delaying Dark-Induced Leaf Senescence

**DOI:** 10.3390/ijms20246167

**Published:** 2019-12-06

**Authors:** Ze Pan, Lihua Chen, Fei Wang, Wangyang Song, Aiping Cao, Shuangquan Xie, Xifeng Chen, Xiang Jin, Hongbin Li

**Affiliations:** 1 Key Laboratory of Xinjiang Phytomedicine Resource and Utilization of Ministry of Education, College of Life Sciences, Shihezi University, Shihezi 832003, China; panze0623@163.com (Z.P.); chenlhshz@163.com (L.C.); wangfshzu@163.com (F.W.); swywinner@163.com (W.S.); aiping9smile@sina.com (A.C.); xiesq@shzu.edu.cn (S.X.); cxf_cc@shzu.edu.cn (X.C.); 2 Ministry of Education Key Laboratory for Ecology of Tropical Islands, College of Life Sciences, Hainan Normal University, Haikou 571158, China

**Keywords:** *Gossypium hirsutum*, ascorbate oxidase, light responsiveness, leaf senescence, H_2_O_2_

## Abstract

Ascorbate oxidase (AO) plays important roles in plant growth and development. Previously, we reported a cotton *AO* gene that acts as a positive factor in cell growth. Investigations on *Gossypium hirsutum AO* (*GhAO*) family genes and their multiple functions are limited. The present study identified eight *GhAO* family genes and performed bioinformatic analyses. Expression analyses of the tissue specificity and developmental feature of *GhAO*s displayed their diverse expression patterns. Interestingly, *GhAO1A* demonstrated the most rapid significant increase in expression after 1 h of light recovery from the dark. Additionally, the transgenic *ao1-1*/*GhAO1A* *Arabidopsis* lines overexpressing *GhAO1A* in the *Arabidopsis* *ao1-1* late-flowering mutant displayed a recovery to the normal phenotype of wild-type plants. Moreover, compared to the *ao1-1* mutant, the *ao1-1*/*GhAO1A* transgenic *Arabidopsis* presented delayed leaf senescence that was induced by the dark, indicating increased sensitivity to hydrogen peroxide (H_2_O_2_) under normal conditions that might be caused by a reduction in ascorbic acid (AsA) and ascorbic acid/dehydroascorbate (AsA/DHA) ratio. The results suggested that *GhAOs* are functionally diverse in plant development and play a critical role in light responsiveness. Our study serves as a foundation for understanding the *AO* gene family in cotton and elucidating the regulatory mechanism of *GhAO1A* in delaying dark-induced leaf senescence.

## 1. Introduction

Ascorbate oxidase (AO; EC 1.10.3.3), is an enzyme that exists only in plants and fungi [1] and participates in numerous physiological processes, including plant growth and development, responses to environmental stress, and regulation of cellular reduction/oxidation (redox) homeostasis. As a glycoprotein of the blue copper oxidase enzyme family [2,3], AO contains three distinct copper domains [4,5,6,7] that endow the function of oxidizing ascorbic acid (AsA) to monodehydroascorbate (MDHA) and of synchronously catalyzing molecular oxygen reduction to water [6,8]. The expression of *AO* is regulated by complex transcriptional and post-transcriptional controls [9], including induced and reduced expression at the mRNA level by hormones [10,11] and abiotic stresses [11,12,13], respectively.

In plants, AOs perform multiple functional roles. *AO* abundance is relatively high in rapidly growing tissues and young fruits [10,13,14]. Transgenic tomato with suppressed *AO* expression displays high levels of AsA in fruits and improved fruit yield [15,16]. Enhancing *AO* expression in tobacco reduces stomatal aperture [11]. AO decreases the production of dehydroascorbate (DHA) by catalyzing the oxidation of AsA, which in turn regulates cell division and cell cycle [17,18,19]. Overexpression of *AO* has also been utilized as a strategy to downregulate oxygen diffusion in root nodules [20]. Higher or lower AO activity results in increased sensitivity to ozone or enhanced tolerance to salt stress, respectively [11,12,21]. In addition, previous studies have also shown that *AO* may be involved in light responsiveness, with *cis*-elements for light regulation in a known *AO* promoter region, and the suppression or overexpression of *AO* causes late flowering or delayed dark-induced senescence in vitro [2,10,12,22].

Reactive oxygen species (ROS) are regarded as signaling molecules that participate in plant development, programmed cell death (PCD), and environmental stress responses [23,24,25]. The enzymes of the AsA antioxidant system play different roles; ascorbate peroxidase (APX) acts as a ROS-scavenging enzyme that reduces hydrogen peroxide (H_2_O_2_) to water, while AO utilizes AsA as a substrate to reduce O_2_ to water [25,26]. A close link between AO and ROS was reported in cotton, wherein H_2_O_2_ significantly accumulates in *AO*-overexpressing tobacco plants [7], suggesting the potential role of AO in modulating cellular ROS-mediated redox homeostasis. It has been reported that AO plays a crucial role in cell growth by regulating the cellular redox state in connection with ROS, demonstrating that AO is upregulated in rapidly expanding tissues [9,27,28,29], and the overexpression of cotton *Gossypium hirsutum ascorbate oxidase 1* (*GhAO1*) and pumpkin *AO* promotes cell elongation in tobacco [7,30].

Upland cotton (*Gossypium hirsutum*) is one of the most important global economic crops and provides the major material of fibers utilized in the textile industry. Previously, we reported that *APX* is involved in fiber development by regulating H_2_O_2_ homeostasis, and fiber-specific *AO* promotes tobacco cell growth by H_2_O_2_-mediated accumulation [7,25]. In higher plants, a total of three, five, seven, four, and six *AOs* have been identified in *Arabidopsis thaliana*, *Oryza sativa*, *Glycine max*, *Zea mays*, and *Sorghum bicolor*, respectively, with different response patterns to diverse abiotic stresses and development cues [3]. The *AO* gene family members and their functions in cotton, however, are largely unknown. In this study, we performed a genome-wide investigation and expression profiling of the *AO* gene family in *G. hirsutum*. A total of eight *GhAO* genes were identified, and the systematic analyses of chromosomal location, phylogenetic relationship, gene structure, and motif distribution were obtained. *Cis*-element distribution and expression pattern analyses of tissue-specificity and developmental feature suggested that *GhAOs* have diverse functions. Particularly, an expression analysis in the process from dark to light, coupled with a complementary analysis of *GhAO1A* in the *ao1-1 Arabidopsis* mutant, revealed that *GhAO1A* is a functional gene that influences plant flowering and delays dark-induced leaf senescence by regulating AsA-mediated ROS homeostasis. These results provide comprehensive evolutionary and functional information on the *GhAO* gene family and lay the foundation for understanding the function and mechanism of *GhAO1A* in light responsiveness by controlling cellular redox balance.

## 2. Results

### 2.1. Identification and Characterization of AO Gene Family Members in G. hirsutum

**Figure 1:**
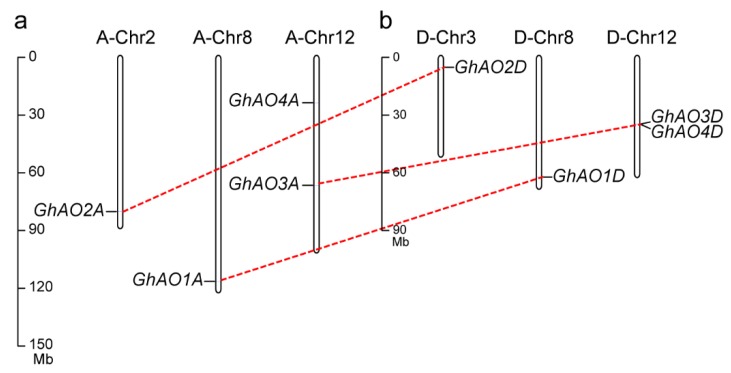
Chromosome distribution of *GhAO* gene family members. The eight *GhAO* genes mapped to the chromosomes of the (**a**) A and (**b**) D sub-genome are shown. Genes were renamed as *GhAO1A-4A* and *GhAO1D-4D*, with previously reported *GhAO1* designated as *GhAO1D*. Different scales were used for chromosomes. Pairs of segmentally duplicated genes are indicated by red dotted lines.

To identify the *AO* gene family members in *G. hirsutum*, the reported AO protein sequences from *A*. *thaliana*, *O*. *sativa*, *G*. *max*, *Z*. *mays*, and *S*. *bicolor* (accession numbers listed in Supplementary Table S1) were used as direct queries to perform a blastp search against the *G*. *hirsutum* protein databases, which identified a total of eight *AO* genes. The open reading frame (ORF) length of these *GhAOs* ranged from 1263 to 1758 base pairs (bp), encoding putative proteins with 420 to 585 amino acids (aa), molecular weights (MW) ranging from 46.61 to 65.64 kDa, isoelectric point (*p*I) ranging from 5.65 to 8.63, and location in the extracellular apparatus (Table 1). The genomic location analysis showed that the eight *GhAO* genes were distributed across six chromosomes of the A or D sub-genome (Figure 1). Three putative paralogous gene pairs (*GhAO1A*-*GhAO1D*, *GhAO2A*-*GhAO2D*, and *GhAO3A*-*GhAO3D*) were found to be segmentally duplicated, sharing more than 98% sequence identity at the nucleotide level. *GhAO3D* and *GhAO4D* showed a possible incidence of tandem duplication due to their presence in proximal positions on the same chromosome.

### 2.2. Phylogenetic and Evolutionary Analyses of GhAO Gene Family

**Figure 2:**
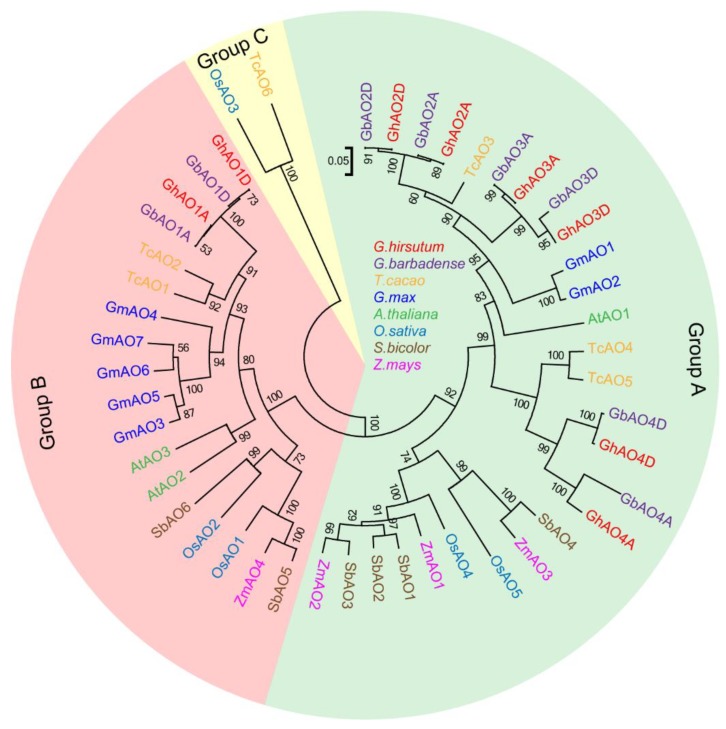
Phylogenetic analysis of *AO* family genes. The AO protein sequences from *Gossypium hirsutum* (GhAOs), *G. barbadense* (GbAOs), *Arabidopsis thaliana* (AtAOs), *Oryza sativa* (OsAOs), *Zea mays* (ZmAOs), *Sorghum bicolor* (SbAOs), *Glycine max* (GmAOs), and *Theobroma cacao* (TcAOs) were used to construct a neighbor-joining (NJ) phylogenetic tree, with a bootstrap of 1000 replicates and illustration of different colors in distinct species.

The AO protein sequences from Gossypium hirsutum (GhAOs), Gossypium barbadense (GbAOs), Theobroma cacao (TcAOs), Arabidopsis thaliana (AtAOs), Oryza sativa (OsAOs), Glycine max (GmAOs), Zea mays (ZmAOs), and Sorghum bicolor (SbAOs) were used to construct a neighbor-joining (NJ) phylogenetic tree. These AOs were divided into three groups (Figure 2), and in terms of the AOs in Malvaceae (cotton and cacao), except for GhAO1A/1D, GbAO1A/1D, TcAO1, and TcAO2 in group B, most of the other AOs were mainly distributed in group A. In addition, based on the distinct differences in protein sequences compared to the other AOs, group C included only two members (OsAO3 and TcAO6).

**Figure 3:**
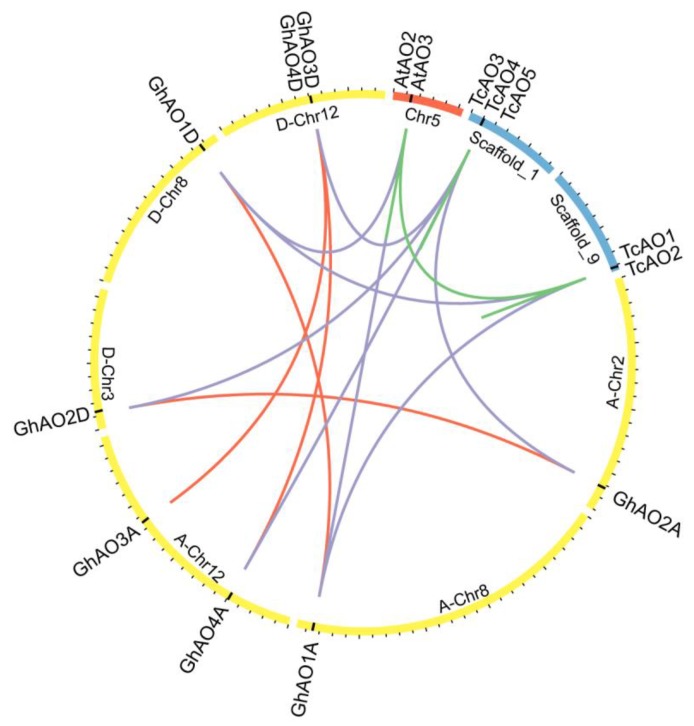
Tandem and segmental duplication of *GhAOs* and syntenic analysis of *G. hirsutum*, *T. cacao*, and *A. thaliana*. Chromosomes and scaffolds from cotton, cacao, and *Arabidopsis* are shown in yellow, blue, and red circular columns, respectively. The positions of the *AO* genes are marked by black lines. Duplicated *GhAOs* are linked by red lines, and syntenic relationships are indicated by purple lines between cotton and cacao or *Arabidopsis* and green lines between cacao and *Arabidopsis*.

The synteny of genes across related plant species could provide insights to their evolutionary relationships. The syntenic analysis of *AO* genes from cotton, cacao, and *Arabidopsis* was performed and visualized using the Circos software. There were four syntenic blocks of *AO* genes containing eight collinear gene pairs between *G. hirsutum* and *T. cacao*, indicating the segmental duplications of *AO* family genes between *G. hirsutum* and *T. cacao* (Figure 3). The Tajima relative rate test was used to determine the evolutionary rate of *G. hirsutum AO* paralogs (Table 2). Notably, an accelerating evolutionary rate was observed in the *GhAO4A*/*GhAO4D* gene pair, suggesting a potential functional divergency of the two *AO* paralogs.

The ratio of nonsynonymous substitution rate/synonymous substitution rate (Ka/Ks) is an indicator of evolutionary selection, with purifying, neutral, and positive selection indicated by K_a_/K_s_ < 1, =1, and >1, respectively. In this study, the Ka/Ks ratio of each pair of duplicated *GhAO* genes indicated that, except for *GhAO4A*/*GhAO4D*, the other three *GhAO* gene pairs were purifying selection, and two of the three duplicated *GhAO* gene pairs were < 1 (Supplementary Table S2), implying that these *GhAO* gene pairs underwent a relatively rapid evolution, thereby influencing the expansion of the *GhAO* gene.

### 2.3. Gene Structure and Motif Analyses of GhAO Genes

**Figure 4:**
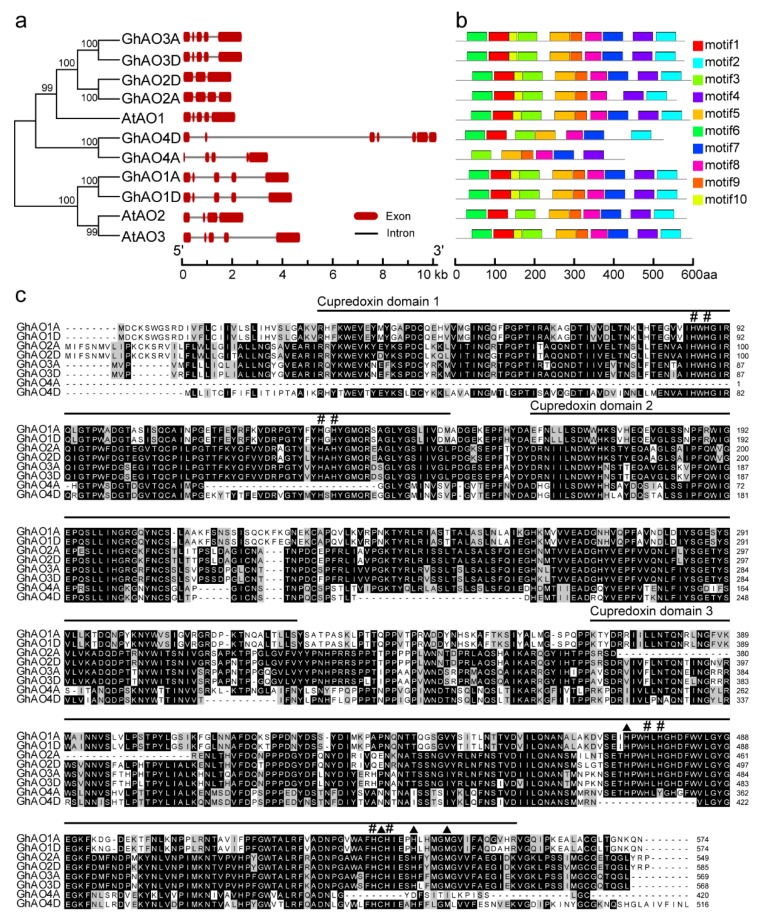
Gene structure and conserved motif analysis of the *AO* gene family in *G. hirsutum* and *A. thaliana*. (**a**) Exons and introns were represented by black lines and red boxes, respectively, with genomic lengths indicated at the bottom. (**b**) Ten conserved motifs distributed in AOs are indicated with different colors, showing the amino acid length at the bottom. (**c**) Amino acid sequence alignment of eight *G. hirsutum* ascorbate oxidase (GhAO) proteins. Black shading indicates strictly conserved residues and gray shading represents regions of less-strict conservation. Three cupredoxin domains are shown. Pounds (#) indicate trinuclear copper binding sites and solid triangles. (▲) represent type 1 (T1) copper binding sites.

Gene structure and conserved motif analyses were performed to obtain more insights into the diversity of motif composition and evolutionary relationship among the *GhAOs.* The arrangement of exons and introns were analyzed by the Gene Structure Display Serve (GSDS) program. Except for *GhAO4A*/*4D*, paralogous *GhAO* gene pairs displayed highly conserved gene structures (Figure 4a). The Multiple Em for Motif Elicitation (MEME) program was used to obtain the conserved motifs in GhAOs, demonstrating that 10 separate motifs were identified in GhAOs and each GhAO contained six or more conserved motifs (Figure 4b). Amino acid sequence alignment of the eight cotton GhAO proteins showed their conserved cupredoxin domains and the enzyme catalytic sites (Figure 4c). These results indicated that GhAOs are conservative proteins sharing similar motif distribution patterns of the same group.

### 2.4. Expression Analysis of GhAO Genes in Fiber Development and Different Tissues

**Figure 5:**
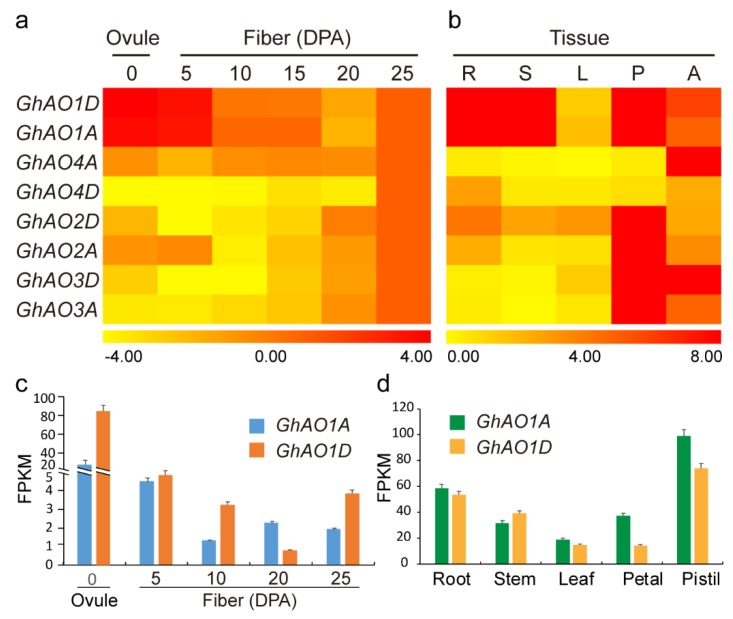
Expression analysis of *GhAOs* in fiber development and different tissues. qRT-PCR-based heatmap was performed to analyze the expression level of *GhAO* genes in (**a**) fiber developmental stages and (**b**) different cotton tissues. R, roots; S, stems; L, leaves; P, petals; A, anthers. *Gossypium hirsutum ubiquitin* 7 (*GhUBQ7*) gene expression was used as the internal control. The qRT-PCR data of 25 days post anthesis (DPA) fibers were utilized for normalization and artificially set to 1 in (**a**). Gene expression abundances are shown with different color scales, with yellow, orange, and red to represent low, moderate, and high expression levels, respectively. RNA-seq data of *GhAO1A* and GhAO1D in (**c**) fiber developmental stages and (**d**) different cotton tissues. The RNA-seq data was obtained from the public released data of the BioProject (PRJNA248163) database of National Center for Biotechnology Information (NCBI). The Fragments Per Kilobase of exon model per Million mapped reads (FPKM) value was calculated by Cufflinks.

To investigate the *GhAO* expression patterns of tissue-specificity and responses to phytohormone and abiotic stresses, a quantitative real-time polymerase chain reaction (qRT-PCR)-based heatmap was used to analyze the expression levels of the *GhAO* genes. The *GhAO* genes exhibited diverse expressions in different tissues, including fibers, roots, stems, leaves, petals, and anthers. During the different fiber developmental stages, *GhAO1A*/*1D* showed high expression levels in 0–15 days post-anthesis (DPA) fibers (initiation and elongation stages), with significant accumulated expression in 0 DPA ovules and 5 DPA fibers. *GhAO4A*/*4D* displayed notable enrichment in 15–25 DPA fibers (secondary wall deposition stage), suggesting that these four *GhAOs* perform diverse important roles in different fiber growth stages. *GhAO1A*/*1D* was predominantly expressed in the roots, stems, petals, and anthers, indicating their potential function in tissue development (Figure 5a). Interestingly, except for *GhAO4A*/*4D* that showed increased expression in anthers, all of the other *GhAOs* demonstrated substantial increased expression in floral organs (Figure 5b). In consideration of the preferential expressions of *GhAO1A*/*1D* in fiber growth and tissue development, the corresponding publicly released RNA-seq data of *GhAO1A*/*1D* was obtained to identify and validate the expression level of *GhAO1A*/*1D*. The highly consistent expression features of *GhAO1A*/*1D* were observed between the qRT-PCR-based heatmap and the RNA-seq data (Figure 5c,d). These results implied that, by the significant accumulations, *GhAO1A*/*1D* may perform a possible vital role in controlling floral tissue development and flowering regulation.

### 2.5. Cis-Regulatory Element Analysis of the GhAO Gene Promoter Region

**Figure 6:**
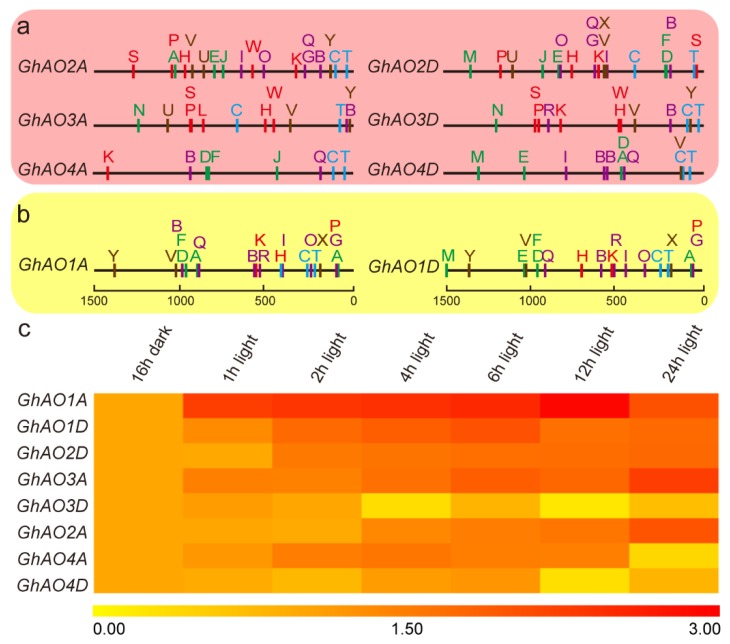
Distribution of *cis*-elements in the *GhAO* promoter regions and expression analysis of *GhAOs* under light recovery from the dark. (**a**,**b**) represent two clades of the phylogenetic tree. The black lines indicate the length of the promoter sequences, with scale bars on the bottom. The colored capital letters represent different *cis*-elements, with blue for core element, green for phytohormone response, red for stress response, purple for light response, and brown for others. Detailed information on each *cis*-element is provided in Supplementary Table S3. (**c**) qRT-PCR-based heat map was performed to analyze the expression pattern of *GhAOs* after light recovery from the dark for 1 h, 2 h, 4 h, 6 h, 12 h, and 24 h. *GhUBQ7* was used as the internal reference gene. The qRT-PCR data of the 16 h dark period was utilized for normalization and artificially set to 1. Results were normalized using *GhUBQ7* as the internal reference. Gene expression abundances are shown with different color scales, with yellow, orange, and red to represent low, moderate, and high expression levels, respectively.

To investigate the regulatory features of *GhAOs*, the isolated 1.5 kb sequence upstream of translational start site of the *GhAOs* was assessed for the presence of *cis*-acting elements. These regulatory elements were composed of various kinds of *cis*-acting elements, including core *cis*-elements and *cis*-elements for phytohormone response, stress response, and light response, and are indicated by capital letters of different colors (Figure 6a,b). The detailed classification, symbol, and sequence information of the *cis*-elements are listed in Supplementary Table S3. Interestingly, diverse light-responsive elements, including G-Box, Box 4, and Box I were found in the promoter regions of all of the *GhAO* genes, which suggested that *GhAOs* may exhibit distinct roles in light-regulated growth and development. In addition, the paralogous genes indicated the relatively conserved distribution of *cis*-elements, inferring the potential relationship of evolution and regulation model for *GhAO* gene pairs. Then, to gain insights on the regulatory mechanism of *GhAO* in response to light, the expression levels of the *GhAOs* were determined during the process of light recovery following continuous treatment in a 16 h dark period. The results showed that the expression of *GhAO1A* was increased significantly after 1 h of light recovery from the dark and then was stably maintained thereafter. Meanwhile, moderate promoted levels of expression of *GhAO1D, GhAO2D*, and *GhAO3A* were observed during light recovery (Figure 6c). These results suggested that *GhAO* genes, particularly *GhAO1A*, that showed the most significant and fastest induced expression during dark to light recovery, may perform pivotal functions in light adaptation and light-regulated growth and development of *G. hirsutum*.

### 2.6. GhAO1A Rescues the Late-Flowering Phenotype of the Arabidopsis ao1-1 Mutant

**Figure 7:**
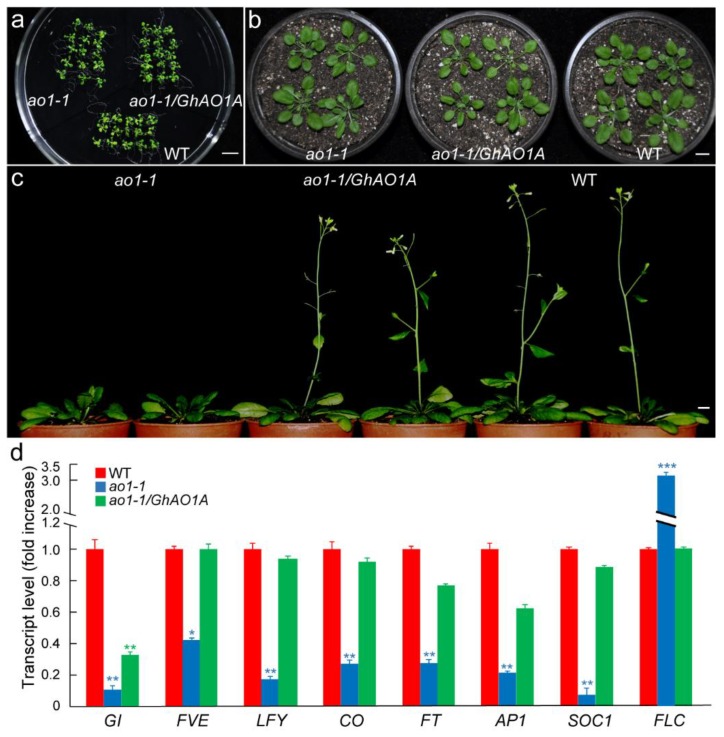
Functional complementary analysis of *GhAO1A* in *Arabidopsis ao1-1* mutant. The phenotypes of *ao1-1* mutant, *ao1-1*/*GhAO1A* transgenic plants, and wild-type (WT) *Arabidopsis* at different growth stages of (**a**) two-week-old seedlings, (**b**) four-week-old seedlings, and (**c**) eight-week-old plants under normal growth conditions (16 h light/8 h dark). Bar = 1 cm. (**d**) qRT-PCR analysis of expression levels of the selected floral genes in eight-week-old *ao1-1* mutant, transgenic *ao1-1*/*GhAO1A*, and WT *Arabidopsis* plants. The results were normalized using *Arabidopsis thaliana 18S ribosomal RNA* (*At18SrRNA*) as the internal reference gene. The gene expression level of WT was artificially set to 1, and was used as the reference for statistical difference analysis. Asterisks indicate significant differences according to one-way ANOVA, * *p* < 0.05; ** *p* < 0.01; *** *p* < 0.001.

Since it has been reported that suppression of *AO* genes alters the vegetative and reproductive growth of plants [12], *Arabidopsis* plants of wild-type (WT), *ao1-1* T-DNA insertion mutants, and *ao1-1*/*GhAO1A* transgenic plants generated by overexpressing *35S::GhAO1A* in the *ao1-1* mutant through genetic transformation by floral dip method were utilized to investigate *GhAO1A* function in controlling plant growth and development in *Arabidopsis*. Under normal growth conditions, no significant phenotype differences in seedlings of the WT, *ao1-1*, and *ao1-1*/*GhAO1A Arabidopsis* plants were observed (Figure 7a,b). After continuous planting, compared to WT, the *ao1-1* mutant exhibited late flowering (Figure 7c). Remarkably, the *ao1-1*/*GhAO1A* transgenic plants showed normal flowering times similar to that observed for the WT plants, indicating that *GhAO1A* is a functional homolog that rescues the defect of delayed flowering in *ao1-1* mutants.

Considering the floral genes’ critical role in controlling flowering development [31,32], the expression of selected genes, namely, *GIGANTEA* (*GI*), *FLOWERING LOCUS VE* (*FVE*), *LEAFY* (*LFY*), *CONSTANS* (*CO*), *FLOWERING LOCUS T* (*FT*), *APETALA 1* (*AP1*), *SUPPRESSOR OF OVEREXPRESSION OF CONSTANS 1* (*SOC1*), and *FLOWERING LOCUS C* (*FLC*), were detected by qRT-PCR. As shown in Figure 7d, except for *FLC* that showed a relatively high expression level in the *ao1-1* mutant, all of the other selected genes accumulated significantly in both transgenic *ao1-1*/*GhAO1A* and WT *Arabidopsis* plants. Most of these genes had similar expression levels in transgenic *ao1-1*/*GhAO1A* and WT *Arabidopsis* plants, with over 2.5-fold enrichment than that observed in the *ao1-1* mutant. These results suggested that *GhAO1A* may affect the flowering development in *Arabidopsis* by altering the expressions of floral genes.

### 2.7. GhAO1A Delays Dark-Induced Leaf Senescence in Arabidopsis

**Figure 8:**
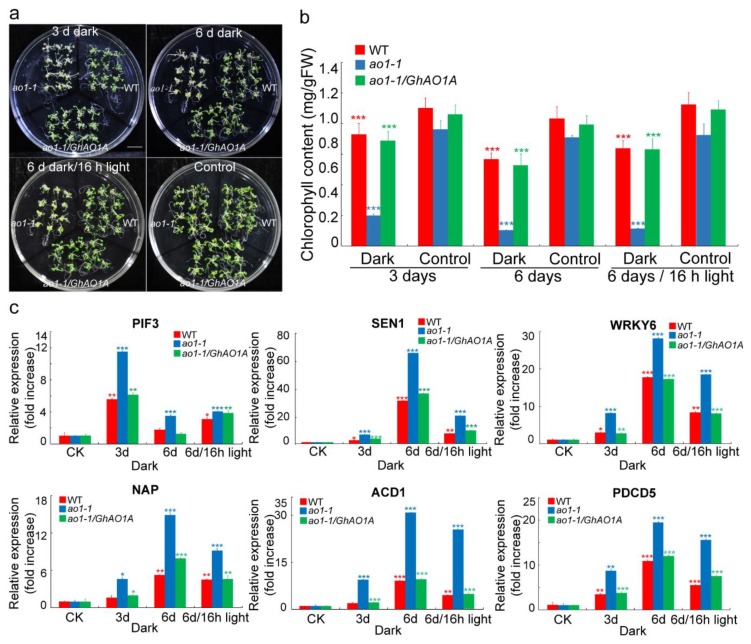
Functional analysis of *GhAO1A* in delaying dark-induced leaf senescence in *Arabidopsis*. (**a**) Representative phenotype of two-week-old *Arabidopsis ao1-1* mutant, transgenic *ao1-1*/*GhAO1A*, and WT plants under normal growth conditions (16 h light/8 h dark), dark treatment, and light recovery (16 h light out of 6 d dark). Bar = 1 cm. (**b**) Chlorophyll content of leaves of two-week-old *Arabidopsis ao1-1* mutant, *ao1-1*/*GhAO1A*, and WT plants. Data are the average of three independent experiments. (**c**) qRT-PCR analysis of senescence-associated genes (SAGs) in the leaves of two-week-old *Arabidopsis ao1-1* mutant, *ao1-1*/*GhAO1A*, and WT plants. The results were normalized using *Arabidopsis thaliana Actin 2* (*AtACTIN2)* as the internal control. Data of untreated *Arabidopsis* plants that were designed as control check (CK) were utilized as references for statistical difference analysis. Asterisks indicate the significant differences according to one-way ANOVA, * *p* < 0.05; ** *p* < 0.01; *** *p* < 0.001.

Transgenic tobacco plants overexpressing *AO* demonstrated a delayed senescence after dark treatment [22]. In this study, two-week-old *Arabidopsis* seedlings (*ao1-1*, *ao1-1*/*GhAO1A*, and WT) were subjected to continuous dark treatment for 3 d and 6 d, and then to light recovery for 16 h to explore *GhAO1A* function in leaf senescence. Macroscopic observation showed the significant senescence phenotype of leaves with yellowing/bleaching or withering in the *ao1-1* mutant, compared to the WT and *ao1-1*/*GhAO1A* transgenic *Arabidopsis* seedlings (Figure 8a). Nearly no significant phenotype difference was observed in the WT and *ao1-1*/*GhAO1A* transgenic seedlings, indicating that *GhAO1A* performs a positive role in delaying dark-induced senescence. In addition, under dark treatment, reduced leaf chlorophyll content was detected in all of the *Arabidopsis* plants, showing an obvious decrease in chlorophyll content with the increase in dark treatment time. Specifically, the leaf chlorophyll content was significantly lower in the *ao1-1* mutant than that in the WT and *ao1-1*/*GhAO1A Arabidopsis* plants. Moreover, when light recovery was implemented, chlorophyll content prominently increased in all of the *Arabidopsis* plants, demonstrating more prompt enhancement in *ao1-1*/*GhAO1A* and WT than that in the *ao1-1* mutant (Figure 8b).

Senescence-associated genes (SAGs) are key factors that induce senescence [33,34]. Altered AO expression results in leaf senescence and finally cell death [35]. The transcript levels of selected genes, namely, *PHYTOCHROME INTERACTING FACTOR 3* (*PIF3*), *SENESCENCE 1* (*SEN1*), *WRKY DNA-BINDING PROTEIN 6* (*WRKY6*), *NAC DOMAIN CONTAINING PROTEIN* (*NAP*), *ACCELERATED CELL DEATH 1* (*ACD1*), and *PROGRAMMED CELL DEATH PROTEIN 5* (*PDCD5*), were analyzed by qRT-PCR. Figure 8c indicates that all of the detected genes exhibited higher accumulation in the *ao1-1* mutant than in the WT and *ao1-1*/*GhAO1A Arabidopsis* plants under dark or light recovery conditions. In the *ao1-1* mutant, except for *PIF3*, that showed peak expression level after 3 d of dark treatment, all of the other selected genes indicated the highest enrichment after 6 d of dark treatment. The expression levels of these SAGs in the *ao1-1*/*GhAO1A* transgenic plants were similar to that in the WT plants. In addition, we also performed an expression analysis for other reported SAGs that exhibited similar expression patterns as the genes evaluated above (Supplementary Figure S1). These results indicated that *GhAO1A* performs an important role in delaying dark-induced leaf senescence possibly induced by SAGs in *Arabidopsis*.

### 2.8. GhAO1A Increases the Sensitivity of Arabidopsis to H_2_O_2_

**Figure 9:**
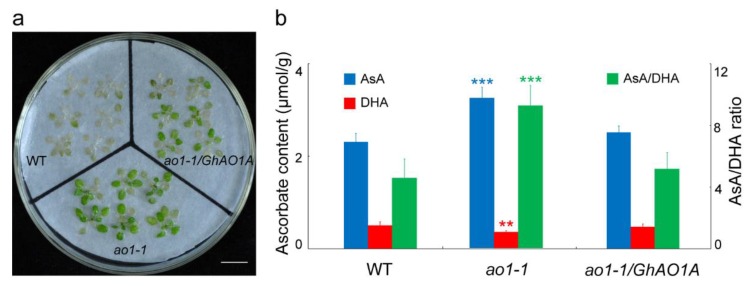
Effect of exogenous hydrogen peroxide (H_2_O_2_) on *Arabidopsis* seedlings. (**a**) Two-week-old *Arabidopsis ao1-1* mutant, transgenic *ao1-1*/*GhAO1A*, and WT plants were incubated with or without 100 mM H_2_O_2_ for two days under normal growth conditions (16 h light/8 h dark). Bar = 1 cm. (**b**) Determination of ascorbic acid (AsA) and dehydroascorbate (DHA) content in *Arabidopsis ao1-1* mutant, transgenic *ao1-1*/*GhAO1A*, and WT plants. The AsA/DHA ratio is also shown. Data are the average of three independent experiments. Results of WT were used as references for analysis of statistical difference test. Asterisks indicate significant differences according to one-way ANOVA, ** *p* < 0.01; *** *p* < 0.001.

H_2_O_2_ is a key factor that causes leaf senescence [36], and based on our previous work reporting that *AO* promotes the oxidative state of tobacco cells through inducing H_2_O_2_ production [7], to further investigate *GhAO1A* function in response to oxidative stress, two-week-old *Arabidopsis ao1-1* mutant, *ao1-1*/*GhAO1A*, and WT seedlings were incubated with 100 mM H_2_O_2_ for two days. The results showed that, after continuous H_2_O_2_ treatment, the *ao1-1* mutants exhibited obvious insensitivity to H_2_O_2_ with less leaves exhibiting bleaching, whereas more green leaves in the *ao1-1*/*GhAO1A* and WT seedlings exhibited withering and bleaching (Figure 9a). Furthermore, in view of the function of *AO* in oxidizing ascorbate [6], the contents of AsA (reduced form) and DHA (oxidized form) were determined to explore the role of *GhAO1A* in controlling the redox state of cells. The results indicated that both the AsA content and the ratio of AsA/DHA significantly increased in the *ao1-1* mutant (Figure 9b). These results showed that *GhAO1A* increases the sensitivity of *Arabidopsis* to H_2_O_2_, which may be caused by promoting oxidation reactions in cells.

## 3. Discussion

### 3.1. Identification and Characteristics of AO Gene Family in Gossypium hirsutum

AO is a member of the multicopper oxidase (MCO) family that catalyzes the redox reactions and plays multiple roles in many physiological processes, including cell elongation with abundant activity in rapid enlarging tissues, retarded flowering development [1,12], and response to environmental stress, through regulation of cellular redox homeostasis by oxidizing the substrate AsA to MDHA and reducing the molecular oxygen to water [3]. *AO* gene family members have been systematically investigated in some plants, including five members in *O. sativa*, three in *A. thaliana*, seven in *G. max*, four in *Z. mays*, and six in *S. bicolor* [3]. In this study, we performed a genome-wide identification of *AO* gene family in *G. hirsutum*, and a total of eight *GhAOs* was identified (Table 1) that were distributed across different chromosomes (Figure 1). Variations in gene family size are a common phenomenon that may be due to gene duplication, deletion, pseudogenization, and/or functional diversification [15]. *AO* genes in cotton and soybean display more duplication events and comprise more family members than other reported crops (Supplementary Table S1, Figure 2). Based on the incomplete assembly of the cotton genome, compared to other *GhAOs*, *GhAO4A*/*D* has an incomplete structure both at the DNA and mRNA levels, as demonstrated by shorter sequence lengths (Table 1, Figure 4).

Plant AOs share typical MCO domains, which endow the enzymatic activity to catalyze redox reactions. The paralogous *GhAOs* located on the A and D chromosomes display similar exon-intron structure and motif distribution, respectively, which are characteristics of the three pre-defined domains (MCO types 1, 2, and 3) that have been identified in plants, suggesting the possible function of AO to trigger the enzymatic reaction using motif 4 as the site for binding copper (Figure 4). Except for the known copper oxidase domains, GhAOs also exhibit some unidentified motifs (motifs 7–10), which are similar to the reported AOs in some plants [3]. A total of 41 AOs from seven plant species were extracted to construct a phylogenetic tree that consisted of three different clades [3]. The phylogenetic tree analysis indicated that GhAO1A/D and other GhAOs were clustered into groups A and B, respectively (Figure 2), indicating their potential diverse functions.

### 3.2. Expression Patterns of AOs Imply Their Diverse Roles in Plant Growth and Development

*AO* genes were reported to display regulated expressions of tissue-specific development (germination, seeding, rosette, bolting, flower, and silique) in many plants. *Arabidopsis AtAO1* and *AtAO2* were upregulated in all of the developmental stages with different expression patterns. *G. max GmAO4* and *GmAO5* indicated high accumulation during shoot growth. *O. sativa AOs* displayed seedling- and flowering-specific higher expression levels [3]. In the current study, the expression patterns of *GhAOs* in different cotton tissues varied, with significant accumulations of *GhAO1A*/*1D* in fibers, roots, stems, petals, and anthers (Figure 5), suggesting that *GhAOs* may exhibit important function in plant growth, especially in flowering development.

Yamamoto et al. demonstrated that the antisense expression of *AO* in tobacco and T-DNA-inserted *Arabidopsis AO* mutant resulted in the late-flowering phenotype [12]. Interestingly, the late-flowering phenotype reverted to normal after transforming cotton *GhAO1A* into the *Arabidopsis ao1-1* mutant (Figure 7), indicating that cotton *GhAO1A* is a functional homolog and plays a crucial role in maintaining regular flowering development. These results showed that *GhAO1A* provides a regulatory mechanism for the increased expression of floral-associated genes and decreased expression of the floral-suppressed gene *FLC* in WT and transgenic *ao1-1*/*GhAO1A Arabidopsis* plants (Figure 7), with the similar result of suppressed expression of the *FLC* gene promoting flowering [37].

### 3.3. Functional Role of AO Genes in Response to Light Regulation

*AO* expression is regulated by light, with significant upregulation of *AO* mRNA by light in tobacco and *Cucurbita pepo* [10,38]. There were six kinds of light-responsive elements in the promoter regions of all of the *GhAOs* (Supplementary Table S3), whereas only the *GhAO1A*/*1D* promoters included all these six kinds of light-responsive elements and the other *GhAOs* contained fewer and incomplete light-responsive elements (Figure 6a,b), coupled with the most fastest accumulation of *GhAO1A*/*1D* transcripts under light recovery from dark (Figure 6c), suggesting the close link between the number of light-responsive elements and the *GhAO* expression levels regulated by light. Additionally, it has been reported that *AO* performs an important function in suppressing leaf senescence induced by dark [22]. The result presented here shows that, in transgenic *ao1-1* mutant plants overexpressing *GhAO1A*, the dark-induced leaf senescence generated by the deletion of *AtAO1* in the *ao1-1 Arabidopsis* mutant was significantly delayed. Meanwhile, inhibited chlorophyll breakdown and suppressed expression of SAGs were also observed in *ao1-1*/*GhAO1A* transgenic *Arabidopsis* lines (Figure 8). This is consistent with the result that altered *AO* expression leads to chlorophyll degradation and cell death [35]. During the leaf senescence process, chlorophyll degradation, chloroplast decomposition (including decrease in starch granules and loosening of thylakoid membrane), and downregulation of photosynthesis-related genes were investigated [39]. Our previous study showed that *AO* expression was regulated by auxin [7,40]. Plant hormones, such as auxin and ethylene that function as a retardant and a reinforcer, respectively, and their crosstalk are the key factors that influence leaf senescence [34,41,42,43]. Thus, it is necessary to perform further investigations on the function of *AO* in leaf senescence in the presence of hormones.

Numerous genes have been reported to be differentially expressed during the leaf senescence process [44], and many SAGs have been verified to be involved in leaf senescence [33,34,45,46,47,48,49]. The detected SAGs of PIFs (*PIF3*, *PIF34,* and *PIF35*), SENs (*SEN1* and *SEN4*), transcription factors (TFs; *WRKY6*, *NAC2,* and *NAP)*, and cell death-related genes (*PDCD5* and *ACD1)*, showed significant abundant accumulation in the *Arabidopsis ao1-1* mutant (Figure 8, Supplementary Figure S1), indicating that the function of *AO* is closely associated with the expression of these genes. In addition, the TFs of NAC and WRKY play important functions in chlorophyll degradation and leaf senescence by binding to *cis*-elements to regulate target gene expression [50]. Many TF-binding sites exist in the promoter region of *GhAOs* (Figure 6, Supplementary Table S3), providing potential regulation sites for the interaction between *GhAO* and TFs in the process of leaf senescence. Moreover, it has been proven that besides NAC and WRKY, other TFs, including MYB and AP2-EREBP are important factors that regulate leaf senescence by participating in hormone signaling pathways [15,49,51,52,53].

### 3.4. Suppressed Expression of AO Increases Arabidopsis Tolerance to H_2_O_2_

Ascorbate metabolism performs an important role in the detoxification of excessive ROS in cells, thus controlling the cellular redox balance, which regulates plant growth and development and cellular response to environmental stresses [54,55]. Light-induced ROS production alters the redox balance of cells in photosynthetic tissues and thus affects plant growth and development [56]. Our previous studies have shown that the ascorbate metabolism enzyme APX plays a significant role in fiber cell elongation by regulating ROS homeostasis, and cotton AO promotes tobacco cell growth through inducing apoplast oxidation [7,25]. As the enzyme oxidizing AsA to generate DHA, AO is a negative factor in ascorbate metabolism to eliminate ROS that is commonly produced under environmental stress [6]. In the study, the *ao1-1* mutant exhibited a more resistant phenotype to H_2_O_2_ and indicated a more reduced state in cells that was caused by a higher AsA/DHA ratio due to AO deletion, compared to WT *Arabidopsis* and the *ao1-1*/*GhAO1A* transgenic lines overexpressing cotton *GhAO1A* in the *ao1-1* mutant (Figure 9). This is consistent with the report that overexpression or suppression of *AO* leads to increased sensitivity or resistance to oxidative stress in tobacco and *Arabidopsis* [11,12]. There is a close correlation between suppressed AO activity and decreased H_2_O_2_ level [12], suggesting the possibility of interaction between *AO* function and ROS. Elucidating the regulatory mechanism of *AO*, including the crosstalk with ROS and TFs, is warranted.

## 4. Materials and Methods

### 4.1. Plant Materials

*A. thaliana* (Columbia ecotype) and *ao1-1* mutant (SALK_108854, obtained from the Arabidopsis Biological Resource Center, Columbus, Ohio, USA) [57] were used in this study and cultured using normal conditions of temperature of 20 ± 2 °C and a photoperiod of 16 h light/8 h dark.

Cotton (*G. hirsutum* L. cv. Xuzhou 142) plants were cultivated in the experimental field of Shihezi University in Shihezi City, China. Different tissues, including roots, stems, leaves, petals, and anthers, were harvested from approximately two-month-old cotton plants. By marking the flowering bolls (artificially defined as 0 DPA), different developmental fiber tissues were collected and stripped from the ovules at 0, 5, 10, 15, 20, 25, and 30 DPA. All of the materials were flash-frozen in liquid nitrogen and stored at −80 °C until use.

Cotton seedlings were grown in a growth chamber under greenhouse conditions of 28 °C under a 16 h light/8 h dark cycle. Six-week-old cotton seedlings were subjected to different light conditions and abiotic stresses, including exposure to 16 h of dark treatment and then to light recovery. All of the treated materials of cotton seedlings and leaves were collected at various time points as previously mentioned, flash-frozen in liquid nitrogen, and stored at −80 °C until analysis.

### 4.2. Identification of AO Gene Family in G. hirsutum

The genomic data of *G. hirsutum* were downloaded from Joint Genome Institute (JGI) (https://phytozome.jgi.doe.gov). To identify the *AO* genes in *G. hirsutum*, previously reported AOs from *A. thaliana*, *O. sativa*, *G. max*, *Z. mays*, and *S. bicolor* were used as queries to perform a local BLAST (blastp, e-value: 1×10^−30^, identify: 50%, score: 400) search against the *G. hirsutum* protein database [3]. Candidate GhAO sequences were analyzed to confirm the presence of the three domains of the cupredoxin family using InterProScan (http://www.ebi.ac.uk/interpro/search/sequence-search) [58]. The AO sequences of *T. cacao* were obtained from JGI (http://phytozome.jgi.doe.gov). The molecular weight (MW) and isoelectric point (*p*I) were calculated by online tools at the ExPASy website (http://web.expasy.org/compute_pi). Subcellular localization was analyzed using the Softberry server (http://linux1.softberry.com). Functional motifs and conserved sites of GhAOs were obtained using the InterProScan software.

### 4.3. Chromosomal Distribution and Duplication Analyses of the GhAO Genes

The MapInspect software was used to visualize the distribution of *GhAOs* on the *G. hirsutum* chromosomes [59]. Duplication events of *AO* genes were determined by multiple sequence alignment. Paralogous *GhAO* gene pairs were identified only when their nucleotide identities were >90%. The K_a_ (nonsynonymous substitution rate) and K_s_ (synonymous substitution rate) values of paralogous genes were estimated by the DnaSP 5.0 software [60], with the K_a_/K_s_ analysis method described elsewhere [61]. The Tajima relative rate test was used to estimate the evolutionary rate. The Circos software was used for collinear analysis [62].

### 4.4. Analyses of Phylogenetic Relationship, Gene Structure, and Motif Distribution

The full-length AO protein sequences of *G*. *hirsutum*, *G. barbadense*, *T*. *cacao*, *A*. *thaliana*, *O*. *sativa*, *G*. *max*, *Z*. *mays*, and *S*. *bicolor* were used to analyze their phylogenetic relationship. These amino acid sequences were aligned using ClustalW [63], followed by phylogenetic tree construction using MEGA5.0 [64] through the NJ method with 1000 bootstrap replicates. The exon-intron structure of the *GhAO* genes was generated using the gene structure display server (GSDS) program [65] by aligning the coding sequences (CDS) with the corresponding genomic sequences. The Multiple Em for Motif Elicitation (MEME) [66] program was used for identification of conserved motifs in AO proteins of *G. hirsutum*.

### 4.5. RNA Extraction, Expression Detection, and Promoter Analysis of the GhAO Genes

Total RNA was extracted from different tissues (fibers at different development stages, roots, stems, leaves, petals, and anthers) using the RNAprep Pure Plant Kit (Tiangen, Beijing, China). First-strand cDNA was synthesized from 1 μg of total RNA using the TIANScript RT Kit (Tiangen, Beijing, China), and then used as a template for qRT-PCR analysis using gene-specific primers (Supplementary Table S4), with the *Gossypium hirsutum ubiquitin 7* (*GhUBQ7*) gene as the internal control [7,67].

qRT-PCR was conducted using SYBR Premix Ex Taq (Tli RNaseH Plus) (Takara, Kusatsu, Japan) with three replicates on the LightCycler 480 II System (Roche, Rotkreuz, Switzerland). The heatmap was generated with the software MultiExperiment Viewer (MeV) as described elsewhere [68] based on the qRT-PCR data. A 1.5 kb region located upstream of the translational start site of each *GhAO* gene was submitted to the online PlantCARE software [69] to predict putative *cis*-elements. The RNA-seq data were obtained from the public released data of the BioProject (PRJNA248163) database of NCBI at the website of https://www.ncbi.nlm.nih.gov/bioproject/. The Fragments Per Kilobase of exon model per Million mapped reads (FPKM) value was calculated by Cufflinks (version 2.1.1, University of Washington, Seattle, WA, USA) [70].

### 4.6. Vector Construction and Arabidopsis Transformation

The amplified full-length CDS of *GhAO1A* containing double enzyme sites of *Bam*H I and *Sal* I was inserted into the vector pCAMBIA2300 to generate the overexpression vector *35S::GhAO1A* that was then transformed into *ao1-1* mutant *Arabidopsis* with *Agrobacterium*-mediated (strain GV3101) floral dip method [71], thereby producing the transgenic *Arabidopsis* lines of *ao1-1*/*GhAO1A*. The obtained homozygous *T_3_* generation transgenic *Arabidopsis* lines generated by screening and selfing were used in the subsequent analyses.

### 4.7. Functional Analysis of GhAO1A in Arabidopsis

Two-week-old *Arabidopsis* seedlings (*ao1-1*, *ao1-1*/*GhAO1A*, and WT) were incubated with 100 mM H_2_O_2_ for two days under normal growth conditions (ddH_2_O as mock control) [12]. AsA and DHA levels were determined as previously described [7]. A dark-induced senescence assay was performed where two-week-old *Arabidopsis* seedlings were subjected to continuous dark treatment for 3 d and 6 d and then light recovery for 16 h. Chlorophyll content determination in *Arabidopsis* seedlings were conducted as described elsewhere [31,72]. The expression levels of flowering-related genes such as *GI*, *FVE*, *LFY*, *CO*, *FT*, *AP1*, *SOC1*, and *FLC;* and SAGs, including PIFs (*PIF3*, *PIF4,* and *PIF5*), SENs (*SEN1* and *SEN4*), *SAG20*, TFs (*WRKY6*, *NAP*, and *NAC2*), cell-programmed death genes (*ACD1* and *PDCD5*), and *PHENOPHYTIN PHEOPHORBIDE HYDROLASE* (*PPH*), were determined by qRT-PCR using *Arabidopsis thaliana 18S ribosomal RNA* (*At18SrRNA*) and *Arabidopsis thaliana Actin 2* (*AtACTIN2*) genes as reference genes to normalize the qRT-PCR data, with the gene-specific primers listed in Supplementary Table S4. The analysis of statistical difference test was performed according to one-way ANOVA.

## 5. Conclusions

We identified eight *AO* gene family members in *G. hirsutum* through a genome-wide investigation and characterized their phylogenetic relationship, evolution and synteny, exon-intron structure, and conserved motif distribution. Expression analyses of *GhAO* genes suggested their potential diverse functions in plant growth and development, especially in flower development and light responsiveness. *GhAO1A* was significantly accumulated after 1 h of light recovery from continuous dark treatment, and recovered the late flowering phenotype of *Arabidopsis ao1-1* mutant to the normal phenotype of WT. Compared to the *ao1-1* mutant, the *ao1-1*/*GhAO1A* transgenic *Arabidopsis* lines generated by ectopic overexpression of *GhAO1A* in the *Arabidopsis ao1-1* mutant exhibited delayed dark-induced leaf senescence and increased sensitivity to H_2_O_2_ under normal conditions, with cells showing higher levels of oxidation, which might be caused by a decrease in both AsA content and AsA/DHA ratio. Our results provide a comprehensive analysis of the *AO* gene family in *G. hirsutum* and suggest a potential regulatory mechanism in which *GhAO1A* delays dark-induced leaf senescence by controlling the ROS-mediated cellular redox balance.

## Figures and Tables

**Table 1:** The *Gossypium hirsutum ascorbate oxidase* (*GhAO*) genes and properties of the deduced proteins.

Gene Name	Gene ID	Chromosomal Location	ORF (bp)	Protein ^a^			
				Size (aa)	MW (kD)	*p*I	Subcellular Localization ^b^
*GhAO1A*	Gh_A08G1967	A-Chr8	1725	574	64.06	8.63	E
*GhAO1D*	Gh_D08G2139	D-Chr8	1725	574	64.12	7.99	E
*GhAO2A*	Gh_A02G1327	A-Chr2	1650	549	61.87	7.05	E
*GhAO2D*	Gh_D03G0441	D-Chr3	1758	585	65.64	7.33	E
*GhAO3A*	Gh_A12G0933	A-Chr12	1710	568	64.18	7.08	E
*GhAO3D*	Gh_D12G0952	D-Chr12	1707	569	64.12	8.07	E
*GhAO4A*	Gh_A12G0719	A-Chr12	1263	420	46.61	5.65	E
*GhAO4D*	Gh_D12G0953	D-Chr12	1551	516	58.07	5.95	E

^a^ The molecular weight (MW) and isoelectric point (*p*I) were calculated by ExPASy (http://cn.expasy.org/tools). ^b^ Subcellular localization was analyzed using Softberry server. E, extracellular (secreted).

**Table 2:** Tajima relative rate tests of *AO* gene pairs in *G. hirsutum.*

Testing Group	Mt ^a^	M1 ^b^	M2 ^c^	X^2^	*P* ^d^
*GhAO1A*/*GhAO1D* with *TcAO1*	438	1	2	0.33	0.5637
*GhAO2A*/*GhAO2D* with *TcAO3*	467	4	7	0.82	0.3657
*GhAO3A*/*GhAO3D* with *TcAO3*	473	3	7	1.6	0.2059
*GhAO4A*/*GhAO4D* with *TcAO5*	268	29	13	6.1	0.0136

^a^ Mt indicates the sum of the identical sites in all three sequences tested, ^b^ M1 represents the number of unique differences in the first paralog, ^c^ M2 shows the number of unique differences in the second paralog, and ^d^ *p* < 0.05 infers that one of the two duplicates has an accelerated evolutionary rate.

## Supplementary Materials

Supplementary materials can be found at https://www.mdpi.com/1422-0067/20/24/6167/s1, Figure S1: Analysis of expression level of selected senescence-associated genes in *Arabidopsis*. qRT-PCR analysis was performed to detect the expression level of the senescence-associated genes in leaves of two-week-old *Arabidopsis* plants of *ao1-1* mutant, *ao1-1*/*GhAO1A*, and WT. Results were normalized using *AtACTIN2* gene as the internal control. qRT-PCR data of control check (CK) were artificially set to 1 and used as references for statistical difference analysis, Asterisks indicate the significant differences according to one-way ANOVA, * *p* < 0.05; ** *p* < 0.01; *** *p* < 0.001, Table S1: List of AO information for database search and evolutionary analysis, Table S2: The K_a_/K_s_ ratios for duplicate *AO* genes in *G. hirsutum*, Table S3: Putative *cis*-elements in the promoter regions of *GhAO* genes, Table S4: Primers used in this work.

## Abbreviations

aa	Amino acid
ACD1	Accelerated cell death 1
AO	Ascorbate oxidase
AP1	APETALA 1
AP2-EREBP	APETALA 2/ethylene-responsive element binding protein
APX	Ascorbate peroxidase
AsA	Ascorbic acid
bp	Base pair
CDS	Coding sequence
CK	Control check
CO	CONSTANS
DHA	Dehydroascorbate
DPA	Days post anthesis
FLC	FLOWERING LOCUS C
FPKM	Fragments Per Kilobase of exon model per Million mapped reads
FT	FLOWERING LOCUS T
FVE	FLOWERING LOCUS VE
GI	GIGANTEA
GSDS	Gene Structure Display Server
Ka	Nonsynonymous substitution rate
Ks	Synonymous substitution rate
LFY	LEAFY
MCO	Multicopper oxidase
MDHA	Monodehydroascorbate
MEME	Multiple Em for Motif Elicitation
MeV	MultiExperiment Viewer
MW	Molecular weight
NAC2	NAC DOMAIN CONTAINING PROTEIN 2
NAP	NAC DOMAIN CONTAINING PROTEIN
NJ	Neighbor-joining
ORF	Open reading frame
PCD	Programmed cell death
PDCD5	PROGRAMMED CELL DEATH PROTEIN 5
PPH	PHENOPHYTIN PHEOPHORBIDE HYDROLASE
*p*I	Isoelectric point
PIF	PHYTOCHROME INTERACTING FACTOR
qRT-PCR	Quantitative real-time polymerase chain reaction
rRNA	Ribosomal RNA
redox	Reduction/oxidation
RT-PCR	Reverse transcription polymerase chain reaction
ROS	Reactive oxygen species
SAG	Senescence-associated gene
SEN	SENESCENCE
SOC1	SUPPRESSOR OF OVEREXPRESSION OF CONSTANS 1
TF	Transcription factor
WRKY6	WRKY DNA-BINDING PROTEIN 6
WT	Wild-type
