# Supplementary material for: Genome-Wide Identification and Expression Analysis of the Ascorbate Oxidase Gene Family in Gossypium hirsutum Reveals the Critical Role of GhAO1A in Delaying Dark-Induced Leaf Senescence
Source: Int J Mol Sci. 2019 Dec 6;20(24):6167. doi: 10.3390/ijms20246167 (PMC6940856; doi:10.3390/ijms20246167)
Supplement: Supplementary file 1 [file ijms-20-06167-s001.zip › Supplementary files_Resubmit_Proof/Supplementary Figures S1_resubmit_proof.doc]

**Supplementary Figure S1.** Analysis of expression level of selected senescence-associated genes (*SAGs*) in *Arabidopsis*. qRT-PCR analysis was performed to detect the expression level of the *SAGs* in leaves of two-week-old *Arabidopsis* plants of *ao1-1* mutant, *ao1-1/GhAO1A* and WT. Results were normalized using *AtACTIN2* gene as internal control. qRT-PCR data of control check (CK) were artificially set to 1 and used as references for statistical difference analysis. Asterisks indicate the significant differences according to One-way ANOVA, **p* < 0.05; ***p* < 0.01; ****p* < 0.001.
