# Supplementary material for: Genome-Wide Identification and Expression Analysis of the Ascorbate Oxidase Gene Family in Gossypium hirsutum Reveals the Critical Role of GhAO1A in Delaying Dark-Induced Leaf Senescence
Source: Int J Mol Sci. 2019 Dec 6;20(24):6167. doi: 10.3390/ijms20246167 (PMC6940856; doi:10.3390/ijms20246167)
Supplement: Supplementary file 1 [file ijms-20-06167-s001.zip › Supplementary files_Resubmit_Proof/Supplementary Tables S1-S4 all_resubmit.doc]

Supplementary Table S1. List of AO information for database search and evolutionary analysis

| **Species** | **Gene name** | **Gene ID** | **Chromosomal location** | **CDS sequence** | **Polypeptide** |
| --- | --- | --- | --- | --- | --- |
|  |  |  | **ChrNo. (Orientation): start-end ^a^** | **(bp)** | **(aa)** |
| *Gossypium barbadense*^b^ | *GbAO1A* | Gbar_A08G021230 | ChrA08(-):113152285-113157298 | 1725 | 574 |
|  | *GbAO2A* | Gbar_A02G014460 | ChrA02(+):91555541-91557453 | 1671 | 586 |
|  | *GbAO3A* | Gbar_A12G010120 | ChrA12(+):67161121-67163729 | 1710 | 569 |
|  | *GbAO4A* | Gbar_A12G010110 | ChrA12(+):66796789-66799664 | 1209 | 402 |
|  | *GbAO1D* | Gbar_D08G021990 | ChrD08(-):59635555-59640705 | 1725 | 574 |
|  | *GbAO2D* | Gbar_D03G004310 | ChrD03(+):5897290-5899422 | 1758 | 585 |
|  | *GbAO3D* | Gbar_D12G010050 | ChrD012(-):32536409-32538750 | 1653 | 550 |
|  | *GbAO4D* | Gbar_D12G010060 | ChrD012(-):32615028-32625217 | 1620 | 539 |
| *Theobroma cacao*^b^ | *TcAO1* | Thecc1EG042023t1 | scaffold_9 (-): 40635105-40639853 | 1698 | 565 |
|  | *TcAO2* | Thecc1EG042024t1 | scaffold_9 (-): 40640009-40645099 | 1728 | 575 |
|  | *TcAO3* | Thecc1EG001179t1 | scaffold_1 (+): 5666503-5669541 | 1740 | 579 |
|  | *TcAO4* | Thecc1EG001174t1 | scaffold_1 (+): 5648803-5652533 | 1722 | 573 |
|  | *TcAO5* | Thecc1EG001176t1 | scaffold_1 (+): 5657147-5660235 | 1818 | 605 |
|  | *TcAO6* | Thecc1EG000551t1 | scaffold_1 (-): 2281111-2285425 | 1617 | 538 |
| *Arabidopsis thaliana*^b^ | *AtAO1* | AT4G39830 | Chr4 (+): 18478929-18481343 | 1749 | 582 |
|  | *AtAO2* | AT5G21100 | Chr5 (+): 7168184-7170928 | 1722 | 573 |
|  | *AtAO3* | AT5G21105 | Chr5 (+): 7172687-7177841 | 1767 | 588 |
| *Oryza sativa*^b^ | *OsAO1* | LOC_Os06g37080 | Chr6 (+): 21893247-21898264 | 1746 | 581 |
|  | *OsAO2* | LOC_Os06g37150 | Chr6 (+): 21951199-21956670 | 1902 | 633 |
|  | *OsAO3* | LOC_Os07g02810 | Chr7 (+): 1055140-1058131 | 1638 | 545 |
|  | *OsAO4* | LOC_Os09g20090 | Chr9 (-): 12029912-2035268 | 1734 | 577 |
|  | *OsAO5* | LOC_Os09g32952 | Chr9 (+): 19662808-19665268 | 1725 | 574 |
| *Glycine max*^b^ | *GmAO1* | Glyma05g33470 | Chr05 (+): 38106684-38109689 | 1734 | 577 |
|  | *GmAO2* | Glyma08g14730 | Chr08 (+): 10724343-10727358 | 1347 | 448 |
|  | *GmAO3* | Glyma13g03650 | Chr13 (-): 3663820-3672157 | 1731 | 576 |
|  | *GmAO4* | Glyma14g04530 | Chr14 (+): 3114029-3120767 | 1746 | 581 |
|  | *GmAO5* | Glyma20g12150 | Chr20 (-): 17069647-17077629 | 1728 | 575 |
|  | *GmAO6* | Glyma20g12220 | Chr20 (-): 17148230-17152571 | 1725 | 574 |
|  | *GmAO7* | Glyma20g12230 | Chr20 (-): 17209500-17214632 | 1329 | 442 |
| *Zea mays* | *ZmAO1* | GRMZM2G064106_T01 | Chr7 (-): 99563273-99566704 | 1710 | 569 |
|  | *ZmAO2* | GRMZM2G163535_T01 | Chr7 (-): 99659271-99672152 | 1725 | 574 |
|  | *ZmAO3* | GRMZM2G386170_T01 | Chr8 (-): 26588462-26590255 | 1746 | 581 |
|  | *ZmAO4* | GRMZM2G141376_T01 | Chr9 (+): 78552685-78557854 | 1743 | 580 |
| *Sorghum bicolor* | *SbAO1* | Sobic.002G183600 | Chr2 (-): 56598065-56600282 | 1713 | 570 |
|  | *SbAO2* | Sobic.002G183700 | Chr2 (-): 56646215-56651762 | 1716 | 571 |
|  | *SbAO3* | Sobic.002G183800 | Chr2 (-): 56654083-56658071 | 1740 | 579 |
|  | *SbAO4* | Sobic.003G014600 | Chr3 (+): 1314659-1316756 | 1764 | 587 |
|  | *SbAO5* | Sobic.010G171800 | Chr10 (+): 50424294-50428647 | 1737 | 578 |
|  | *SbAO6* | Sobic.010G171900 | Chr10 (-): 50443758-50447186 | 1917 | 638 |

^a^ ChrNo. (Orientation): start-end: “+” and “-” indicate the forward and reverse orientation, respectively. ^b^ The *AOs* of the five species were used for database search.

Supplementary Table S2. The Ka/Ks ratios for duplicate *AO* genes in *G. hirsutum*

| Paralogous Genes | Ka | Ks | Ka/Ks | Selective pressure |
| --- | --- | --- | --- | --- |
| *GhAO1A – GhAO1D* | 0.0083 | 0.032 | 0.2594 | Purifying selection |
| *GhAO2A – GhAO2D* | 0.0118 | 0.0389 | 0.3033 | Purifying selection |
| *GhAO3A – GhAO3D* | 0.0126 | 0.0311 | 0.4051 | Purifying selection |

Ka: nonsynonymous substitution rate; Ks: synonymous substitution rate.

Supplementary Table S3. Putative *cis*-elements in the promoter regions of *GhAO* genes

| **Classification** | **Symbol** | ***cis*-element** | **Sequence *** | **Description** |
| --- | --- | --- | --- | --- |
| Transcription initiation | T | TATA box | TATAAAT | Core promoter element around -30 of transcription start |
|  | C | CAAT box | CCAAT | Common *cis*-acting element in promoter and enhancer regions |
| Phytohormone response | A | ABRE | GCAACGTGTC | *cis*-acting regulatory element involved in ABA responsiveness |
|  | D | CGTCA-motif | CGTCA | *cis*-acting regulatory element involved in the MeJA-responsiveness |
|  | E | ERE | ATTTCAAA | Ethylene-responsive element |
|  | F | TGACG-motif | TGACG | *cis*-acting regulatory element involved in the MeJA-responsiveness |
|  | J | TCA-element | GAGAAGAATA | *cis*-acting element involved in salicylic acid responsiveness |
|  | M | TGA-element | AACGAC | Auxin-responsive element |
|  | N | TATC-box | TATCCCA | *cis-*acting element involved in gibberellin-responsiveness |
| Stress response | K | TC-rich repeats | ATTTTCTTCA | *cis*-element involved in defense and stress responsiveness |
|  | H | HSE | AAAAAATTTC | *cis-*acting element involved in heat stress responsiveness |
|  | L | LTR | CCGAAA | *cis*-acting element involved in low-temperature responsiveness |
|  | P | Box-W1 | TTGACC | Fungal elicitor responsive element |
|  | S | MBS | TAACTG | MYB binding site involved in drought-inducibility |
|  | W | WUN-motif | TCATTACGAA | Wound-responsive element |
| Light response | R | as-2-box | GATAATGATG | Involved in shoot-specific expression and light responsiveness |
|  | G | G-Box | CACGTT | *cis*-acting regulatory element involved in light responsiveness |
|  | Q | G-box | TAAACGTG | *cis-*acting regulatory element involved in light responsiveness |
|  | B | Box 4 | ATTAAT | Part of a conserved DNA module involved in light responsiveness |
|  | O | Box I | TTTCAAA | Light responsive element |
|  | I | I-box | ATGATATGA | Part of a light responsive element |
| Others | V | circadian | CAANNNNATC | *cis*-acting regulatory element involved in circadian control |
|  | X | CAT-box | GCCACT | *cis*-acting regulatory element related to meristem expression |
|  | Y | ARE | TGGTTT | *cis*-acting regulatory element essential for the anaerobic induction |
|  | U | 5`UTR Py-rich stretch | TTTCTTCTCT | *cis-*acting element conferring high transcription levels |

*. N = A, C, G or T.

Supplementary Table S4. Primers used in this work

| **Primer Name** | **Primer sequence^a^** | **Product size (bp)** |
| --- | --- | --- |
| **For plasmid construct** | | |
| *GhAO1* sense | 5'-CGGGATCCATGGATTGTAAATCTTGGGGTT-3' | 1722 |
| *GhAO1* anti-sense | 5'-ACGCGTCGACGTTCTGCTTGTTTCCTGTTAAT-3' |  |
| **For qPCR analysis** | | |
| *GhAO1A* sense | 5'-AATCCCCCCCAGACAACTA-3' | 270 |
| *GhAO1A* anti-sense | 5'-TTCCTTAATGGCGGGTTC-3' |  |
| *GhAO1D* sense | 5'-CACCATTAAGAAACACTGC-3' | 152 |
| *GhAO1D* anti-sense | 5'-CACGATGGGCATCTTGAGC-3' |  |
| *GhAO2A* sense | 5'-GCCGAACACCTGGTCCTA-3' | 171 |
| *GhAO2A* anti-sense | 5'-CCGGGTAAAATCGGACATT-3' |  |
| *GhAO2D* sense | 5'-GCGGAACACCAGGTCCTA-3' | 171 |
| *GhAO2D* anti-sense | 5'-CCTGGCAAAATCGGACATT-3' |  |
| *GhAO3A* sense | 5'-TCCAGGTCAGGGAGTTTTGG-3' | 185 |
| *GhAO3A* anti-sense | 5'-TTGAGGAAAACTATGACCC-3' |  |
| *GhAO3D* sense | 5'-TGGTCAGGGCGTTTTGGTT-3' | 185 |
| *GhAO3D* anti-sense | 5'-TTAAGGAAGACTATGACCC-3' |  |
| *GhAO4A* sense | 5'-GTTGGTCCTTAAACAACGTTTCC-3' | 157 |
| *GhAO4A* anti-sense | 5'-GCATTTGTGTTGTTTGCAACACT-3' |  |
| *GhAO4D* sense | 5'-ACAACATTTCCCATACCCTTCC-3' | 267 |
| *GhAO4D* anti-sense | 5'-CATGCCCATGAAGATGCCAA-3' |  |
| *GhUBQ* sense | 5'-GAAGGCATTCCACCTGACCAAC-3' | 198 |
| *GhUBQ* anti-sense | 5'-CTTGACCTTCTTCTTCTTGTGCTTG-3' |  |
| *AtACD1*-sense | 5'-ACGGCATGGTAAGAGTCAGC-3' | 249 |
| *AtACD1-*anti-sense | 5'-AAACCAGCAAGAACCAGTCG-3' |  |
| *AtWRKY6*-sense | 5'-TAGTCACGACGGGATGATGA-3' | 142 |
| *AtWRKY6*-anti-sense | 5'-ATTAGGAGGCGGAGGTGAGT-3 |  |
| *AtNAP-*sense | 5'-GCCATTCACAGCGGTTCAAG-3' | 240 |
| *AtNAP-*anti-sense | 5'-TTCGTCCATGAAACCCTCTTG-3' |  |
| *AtSEN1-*sense | 5'-AACATGTGGATCTTTCAAGTGCC-3' | 107 |
| *AtSEN1-*anti-sense | 5'-CTCTGCTTGTTGTCGTTGCTT-3' |  |
| *AtPDCD5-*sense | 5'-GCAAGAGAAACAGCAGGAAGA-3' | 158 |
| *AtPDCD5-*anti-sense | 5'-CCCTCAAAATAACATCCTCCA-3' |  |
| *AtPIF3-*sense | 5'-GGTATGGGAATGCCTTATGCA-3' | 150 |
| *AtPIF3-*anti-sense | 5'-GGCAAAGATACCACCACCAGA-3' |  |
| *AtPIF4-*sense | 5'-GCAAGTTCCTCATCAGGTGGC-3' | 232 |
| *AtPIF4-*anti-sense | 5'-TATCTCTCCTCCTCCTTTCGG-3' |  |
| *AtPIF5-*sense | 5'-TCTCCTGGATACAATACCCTC-3' | 222 |
| *AtPIF5-*anti-sense | 5'-TATTTGTTGACTGGTTGCTGC-3' |  |
| *AtSEN4-*sense | 5'-GTCGGCTGCTGATTTCAACAC-3' | 205 |
| *AtSEN4-*anti-sense | 5'-AAAAGTTGTGACTGTTCCTGC-3' |  |
| *AtSAG20-*sense | 5'- TCGGTAACGTTGTTGCTGGA-3' | 139 |
| *AtSAG20-*anti-sense | 5'- ACCAAACTCTTTCAAATCGCCA-3' |  |
| *AtNAC2-*sense | 5'- TGGAAGACGACTGGGAAAGAT-3' | 240 |
| *AtNAC2-*anti-sense | 5'- ACCATACTGCTCCCCATTCTT-3' |  |
| *AtPPH-*sense | 5'- CAATCATGCTTGCTCCTGGTG-3' | 162 |
| *AtPPH-*anti-sense | 5'- AGTATGGAGCGTTGGGGATTT-3' |  |
| *AtACTIN2-*sense | 5'-CGGTAACATTGTGCTCAGTGG-3' | 222 |
| *AtACTIN2-*anti-sense | 5'-CCTGGACCTGCCTCATCATAC-3' |  |

^a^ Restriction enzymatic sites of *Bam*H Ι and *Sal* Ι are shown in underline.
